# Community acceptance, satisfaction, and support for case management of malaria of various degrees in selected rural communities in Ibadan, Oyo-State

**DOI:** 10.4314/gmj.v55i3.4

**Published:** 2021-09

**Authors:** IkeOluwapo O Ajayi, Ayodele S Jegede, Akintayo O Ogunwale, Janet Ogundairo, Oladipupo S Olaleye, Frederick O Oshiname, Catherine O Falade

**Affiliations:** 1 Department of Epidemiology and Medical Statistics, Faculty of Public Health, College of Medicine, University of Ibadan, Nigeria; 2 Department of Sociology, Faculty of Social Sciences, University of Ibadan, Nigeria; 3 Department of General Studies, Oyo State College of Agriculture and Technology, Igboora, Oyo State, Nigeria; 4 Department of Health Promotion and Education, Faculty of Public Health, College of Medicine, University of Ibadan, Oyo State, Nigeria; 5 Department of Public Health, School of Public and Allied Health, Babcock, University, Ilishan-Remo, Nigeria; 6 Department of Pharmacology & Therapeutics, College of Medicine, University of Ibadan, Nigeria

**Keywords:** malaria, case management, community health workers, voluntary workers, Nigeria

## Abstract

**Objectives:**

This study aimed to assess communities' perception and adoption of the evidenced-based malaria diagnosis and case management intervention targeted at under-five children. The effectiveness of trained Volunteer Community Health Workers (VCHWs) to diagnose malaria among under-five children using rapid diagnostic testing kit, provide treatment using Artemisinin Combination Therapy and rectal Artesunate were assessed.

**Design:**

A qualitative evaluation study was conducted in October 2015.

**Setting:**

Communities in the 6 rural wards in Ona-Ara Local Government Area, Oyo State Nigeria.

**Participants:**

Caregivers of under-five children, community-based frontline health workers, and community leaders selected using purposively sampling.

**Methods:**

Nine Focus Group Discussions and 15 Key Informant Interviews were conducted using a pre-tested guide. Data were subjected to thematic analysis.

**Results:**

It was disclosed that VCHWs promoted people's access to prompt and appropriate malaria treatment. The communities accepted the VCHWs; the reasons given for this included the following: effectiveness of VCHWs in case management of malaria; good inter-personal relationship with caregivers; and the positive health outcomes associated with services provided by them. In addition, community members expressed satisfaction with the VCHWs and provided them with all the support needed to function throughout the malaria case management intervention. The VCHWs considered the support as a great source of encouragement.

**Conclusions:**

The use of VCHWs to treat malaria was adjudged to be effective and considered acceptable to the communities. The adoption of the intervention and its integration into the primary health system by the government is advocated for in medically underserved rural communities.

**Funding:**

This work was supported by UNICEF/UNDP/World Bank/WHO Special Programme for Research & Training in Tropical Diseases, World Health Organization, Geneva, Switzerland (project ID: A80550 [Nigeria] through funds made available by the European Commission (FP7) for research to improve community access to health interventions in Africa.

## Introduction

Malaria is a leading cause of childhood morbidity and mortality in Nigeria.^1^–^4^ According to the WHO *World Malaria Report* of 2019, Nigeria contributed 24 per cent to the 405,000 malaria related deaths globally.^5^ Malaria is one of the leading causes of death among under-five children in Nigeria.^6^,^7^

For instance, in 2015, the disease accounted for 20 per cent of the under-five mortality in Nigeria.^7^ In the past, chloroquine was the drug of choice for the management of malaria. However, with the upsurge in chloroquine-resistant malaria, the Nigerian government changed the malaria treatment policy in 2015 from chloroquine to Artemisinin-based-Combination Therapy (ACT) involving, for instance, the use of artemether-lumefantrine. This decision was in line with the recommendation by WHO relating to the management of malaria^8^. The WHO also recommends that malaria diagnosis be parasite-based as much as possible before treatment with ACT facilitated by the use of microscopy and/or rapid diagnostic tests.^9^–^11^ In addition, the WHO advocates that rectal artesunate should be used as pre-referral treatment of severe malaria in children aged less than six years; trained community members^12^,^13^ can provide this treatment service. These new recommendations are important public health innovations that have the potentials for evidence-based control of malaria at the community level. The approach is similar to the Community Directed Intervention, which was noted in a previous study^14^ to improve access to malaria treatment, especially in resource-limited communities.

Prompt and appropriate home management of suspected cases of malaria among under-five children have been identified as an effective way of reducing malaria related morbidity and mortality.^11^,^15^ Patent medicine vendors (PMVs) and other categories of community members trained as Voluntary Community Health Workers (VCHWs) have been identified as active participants in the home treatment of malaria.^12^,^13^,^16^ The VCHWs have beneficial roles in helping medically underserved communities access primary health care services, including home management of malaria. The Alma Alta Declaration of 1978 incorporated VCHWs into the delivery of basic health services at the village level^17^; this paved the way for the emergence of several VCHWs programmes.^16^,^18^

It has been demonstrated that trained community volunteers can use Rapid Diagnostic Test (RDTs) to diagnose malaria, ascertain the severity of the case and initiate appropriate Primary Health Care (PHC) interventions.^19^ Many studies support the use of RDTs to confirm malaria diagnosis before initiating treatment involving ACT related medicines or rectal artesunate depending on the level of severity.^20^–^22^ As part of efforts to implement effective community case management of malaria of varying severity in sub-Saharan Africa, a multi-country intervention study was designed and conducted in Nigeria, Burkina Faso and Uganda to determine the feasibility and effectiveness of using VCHWs to treat malaria, especially among the under-five children. To achieve this, VCHWs were recruited and trained to perform the following PHC tasks relating to case management of malaria: recognition of signs and symptoms compatible with acute uncomplicated malaria; recognition of danger signs associated with severe malaria; theoretical and hands-on performance and reading of malaria rapid diagnostic tests; administration of artemether-lumefantrine (Coartem™; Novartis Pharma) to children with acute uncomplicated malaria that test positive to RDT; and administration of rectal artesunate to those with danger signs irrespective of the RDT result. After the training, VCHWs were mobilised and provided with necessary resources and supportive supervision to be involved in the routine case management of malaria among under-five children.

At the end of the afore-mentioned multi-country study, the Nigerian team designed and carried out an evaluative site-specific qualitative study in October 2015. This was aimed at determining the acceptance of VCHWs, community satisfaction with the model malaria case management and the nature of support provided for the VCHWs by communities. This paper focuses strictly on the postintervention qualitative evaluation study results conducted in selected rural communities in Ibadan, Oyo State, Nigeria, that participated in the intervention.

## Methods

### Study Setting

This study was conducted in Ona-Ara LGA, Oyo State, South-West Nigeria. The LGA, characterised by a mixture of rural and peri-urban features, was created in 1989 with Akanran as the administrative headquarters. The climatic conditions of the LGA are similar to the ones that prevail in most communities in tropical rainforest zones, which favour the survival of *Anopheles* mosquitoes, the vectors that transmit *Plasmodium. Plasmodium Falciparum* species is the most common cause of malaria in Nigeria.

The LGA consists mainly of people of the Yoruba ethnic group. The major religions in the LGAs are Christianity, Islam, and Traditional African Religion. The people are predominantly farmers, while a few community residents are petty traders, craftsmen, and hunters. There are two health care systems in the LGA – the formal and informal health care systems. The formal health care system involves operating 20 public health care facilities, including Primary Health Care (PHC)/maternity centres. There is an average of two public health care facilities in each of the ten wards in the LGA manned by nurses/midwives, community health extension workers and dispensers who can manage acute uncomplicated malaria. In addition, there are 14 private health care facilities in the LGA. Thirteen out of these private health facilities are located within the peri-urban areas of the LGA. They are far from any of the study communities, with only one of them within the rural setting of the LGA.

The informal health system is operated by traditional healers/herbalists, PMVs and itinerant drug peddlers who do not receive any form of organised formal training.

### Study design and conduct of the qualitative study

This qualitative evaluation study targeted VCHWs trained to manage malaria of varying severity and key stakeholders in the study communities; these include head of health care facilities, village heads and various categories of other community opinion leaders. The VCHWs were women aged 35 to 65 years who live and work in the rural agricultural communities; some worked as PMVs or traditional birth attendants before their recruitment. Information about the nature of the intervention and the processes of selecting and training the VCHWs have been published elsewhere.^23^ In brief, the intervention was case management of malaria among under-five children with varying degrees of severity implemented in 33 villages in the study area for one year. It involved the provision of care to 2,148 under-five children through the activities of 55 trained VCHWs.

The core eligibility criteria for recruitment, training and involvement in the malaria treatment programme as a VCHW included the following: being a permanent resident of a community in the study area; being a nominee of a community; willingness to participate in the programme and willingness to serve the community that has nominated one with honesty and dedication; and willingness to be involved in the part-time assignment without any monetary rewards. The participants were informed right from the onset that altruism was a key philosophical principle that underpins involvement in the programme as a VCHW.

During the pre-service training for the recruited volunteers, the place of altruism in the service of the community as a volunteer was again discussed and stressed. In addition, the voluntary nature of involvement in the programme, the roles and responsibilities of a VCHW, as well as the fundamental ethical principles that must be observed while relating with clients were discussed.

The VCHWs were encouraged to carry on with their volunteer malaria treatment services and their primary occupations, which serve as their means of livelihood without conflict of interest. Parents/guardians of sick children took them to the VCHWs home and informed them about the sickness of their children. The VCHWs responded by examining such sick children for uncomplicated illness signs and treating them using ACTs when the RDT result is positive.

Under-five children with severe malaria cases or danger signs were treated with rectal artesunate inserted into the anus without necessarily waiting for RDT result and thereafter urgently referred by VCHWs to PHC facilities based in the communities or the hospitals in Ibadan metropolis for proper care. The VCHWs paid follow-up visits to their clients to monitor the outcome of their care and provide necessary counselling services. For children who were RDT negative and did not have danger signs, paracetamol was administered in the presence of fever. Furthermore, the VCHWs counted the respiratory rate and referred children with an increased respiratory rate (>50 / minute (6–<12 months) or >40 / minute (12–59 months). If the respiratory rate was not increased, such children would be reviewed after 24 hours by the VCHWs.

The two qualitative instruments used to facilitate the conduct of the study were Focus Group Discussions (FGD) and Key Informant Interviews (KII) guides. The FGD guide was designed to target caregivers of under-five children and the VCHWs. On the other hand, the KII guide was developed to interview key stakeholders in the study communities. The two instruments were designed to explore factors that have potential to influence the health programme's adoption and sustainability. The key issues which cut across the instruments included the following: perception of community acceptance of VCHWs; perceptions relating to satisfaction with the services provided by the VCHWs; nature or form of appreciation of the services provided by the VCHWs; and nature of the support for the VCHWs by the communities.

The FGD and KII guides were initially designed in English; then, they were given to a language expert to translate them to Yoruba, the most commonly spoken language in the study communities. The Yoruba versions of the two instruments were given to another experienced language expert to translate back to English.

Three Research Assistants (RAs) who were all university graduates with a wealth of experience in qualitative studies were recruited and trained to conduct the FGDs and KIIs. Before the use of the instruments, both English and Yoruba versions were pre-tested in Ido LGA among individuals who shared similar characteristics with those in the study site. The results of the pre-test and the experiences acquired during the exercise were used to improve the quality of the instruments and the data collection process.

The FGDs were conducted in conducive environments. Each of the FGDs was conducted by the three-person team of hired RAs. One of the RA served as moderator, another played the role of the note-taker/recorder, while the third person functioned as the observer. A total of nine Focus Group Discussions (FGDs) were conducted. The FGDs were conducted as follow: two among VHCWs; two among male caregivers, and five among female caregivers. The number of participants per FGD group ranged from 8 – 9, while the average duration of each FGD was 60 minutes.

A total of 15 KIIs were conducted among various groups of key informants; the categories of key informant interviewees were as follow (with the number per category indicated alongside in parenthesis): village heads (3); community/opinion leaders (2); traditional birth attendant (1); traditional healer/herbalist (1), religious leaders (4); women leader (1) and heads of Primary Health Care Centres (3).

### Data Analysis

Several procedures were involved in the data management and analysis. The process started with the transcription of the FGDs and KIIs tape recordings. The transcribed notes were subjected to validation and the addition of ideas gleaned from non-verbal communication. Inductive-dominant coding approach^24^ was employed in the coding of the data. The codes and specimens of the generated quotes were checked thoroughly and presented to a team of three experienced qualitative researchers for review and critique. Themes were generated based on the following: (a) content of the study instruments (b) sample quotes from transcripts, and (c) group reflections (contributions from members of the research team). After the initial analysis, the study team met to review the results and modifications were made where necessary. The FGDs and KIIs results were triangulated and coherently presented based on the objectives of the study.

### Ethical Considerations

Ethical approval for the study was obtained from the Oyo State Research Ethics Review Committee, Ministry of Health, Oyo State (Ref No: AD 13/479/71). Written Informed consent was obtained from all the participants.

## Results

### Acceptability of the VCHWs

The FGDs revealed that VCHWs were regarded as health workers with reluctance at the initial stage. A major factor responsible for this was that they were not formal health workers such as nurses. Their competence was in doubt, and so caregivers were not willing to entrust the life or health of their under-five children in their hands. The low social status of the VCHWs in the community before their recruitment and training was another factor that contributed to rejection. They were known to be involved in subsistent occupations such as petty trading and the sale of patent medicines. This aspect of their sociodemographic characteristics adversely affected people's trust in them as providers of care; evidence for this can be deduced from the following quotes:

*“At first, majority of the community dwellers were of the opinion that the VCHWs were women known to be orange sellers, PMVs or persons involved in menial jobs in the community before they were recruited and trained. They were, therefore, perceived as incapable of treating children having malaria” (Male caregivers, FGD, Kajola).*

*“Some people looked down on VCHWs because they are not trained to be nurses. They are not midwives and so people are weary of them complicating their children's health problems. People will prefer to go into the bush to collect leaves and roots to make herbal concoctions for their children instead of patronising VCHWs” (Female Caregivers, FGD, Badeku).*

People's perception relating to the competence of the VCHWs became favourable over time during the programme's implementation. This was when it was observed that the VCHWs were treating malaria in underfive children with positive outcomes. The quality of care provided by the VCHWs also contributed to their acceptance as competent providers of care to children with malaria. The indicators of this included the following: a welcoming attitude; provision of good care; and referral of cases, when the need arises, to places where they can be better managed. The quotes that attest to their eventual acceptance include the following:

*“In Kupalo Village, people always to go to Iya ibeji (Mother of twins) who is a VCHW when their children have fever; she always welcomes them and take good care of their children (Female caregiver, FGD, Jago). The acceptability of the VCHWs increased tremendously when it was noted that they could treat malaria cases brought to them and the cases recovered quickly. There is no any problem concerning with the way they manage malaria in under-five. The VCHWs even assist children that need blood by referring them to a place in the city” (Male caregivers, FGD, Kajola).*

### Satisfaction with VCHWs and the community-based malaria treatment programme

The study participants were requested to appraise the programme to indicate whether they were satisfied with it or not. A view that cuts across the FGDs and KIIs participants were that people were satisfied with the services provided by the VCHWs. Key indicators of satisfaction gleaned from the participants' appraisal relate to easy access to malaria treatment in under-five, treatment efficacy, competence and satisfaction with the services of the VCHWs.

*Access to malaria treatment*

According to the participants, the scheme helped to improve caregivers' access to the treatment of simple malaria involving their under-five children. The perceived indicators of access to malaria treatment offered by the VCHWs included approachability and availability most of the time for consultation. The quotations which reflected this perception include the following:

*“The care they are giving to our children is very good. They (VCHWs) move from village to village to attend to our health needs. The VCHWs give us peace of mind” (Female Caregivers, FGD, Akanran).*

*“They (VCHWs) are readily available and approachable; we no longer worry about where to take our children to for care when they fall sick...we no longer worry about going to Ibadan main town which is a far distant place” (Female TBA KII, Badeku).*

*“The way I see this programme is that it is a good one. It has given parents in the town rest of mind unlike before when there was cases of malaria and the children had to be taken to places like Ijebu and Ibadan for treatment. But now, they have the opportunity of accessing the same care in the community. The children are well taken care of” (Christian Leader, Kajola Village, Male).*

*Treatment efficacy and satisfaction with services of VCHWs*

The participants said that communities were happy and satisfied with the services provided by the VCHWs; they were said to be dedicated to their work and that their services were effective and affordable. The honesty displayed by VCHWs and trust in them encourages caregivers to take their children to them for care. The quotations that highlight people's perceptions of the efficacy of the provided treatment and/or satisfaction with the services include the following:

*“Villagers are satisfied with their work; The VCHWs are good at what they are doing. There is no error in their work...and they do not create any problems whatsoever” (Male caregivers, FGD, Badeku).*

*“People are pleased with us. Parents that have benefited from our services are the ones telling others to bring their children to us for care” (VCHWs, FGD Akaran).*

*“Parents take their children to the VCHWs first before coming to the health centre. If parents discover that the VCHWs have provided insufficient treatment they then take sick children to the health centre” (Head PHC centre KII, PHC centre Ojoku, Female).*

*“What I see as their behaviour and attitude is that the VCHWs do not shout on people unlike the way they do in the hospital. All these make us to be satisfied with them. In addition, they have the fear of God with the work they do” (Traditional Healer KII, Badeku, Male).*

### Respect, social support and show of appreciation to the VCHWs

*Respect for the VCHWs*

Participants were unanimous in stating that VCHWs were held in high esteem. The VCHWs were said to have earned the respect of the people as a result of the following professional attributes: good interpersonal relationship with caregivers; reliance on the result of a simple test (i.e. RDT) to establish a case of malaria before treatment; and provision of effective case management of malaria. Typical participants' quotations are as follow:

*“We appreciate the way they are taking care of children in the community and this is why we respect them. The people see the VCHWs as those that are knowledgeable about health and this is contributing to the good health of the children” (Muslim Leader KII, Kajola, Male).*

*“We are pleased with the VCHWs in our community. We honour them and our attitude to them is positive. We see them as ‘Iya ewe’ (i.e. mothers of children) because they attend to us very well and treat our children anytime they have symptoms of malaria. They check the blood and once a child tests positive to malaria they treat with medicines” (Female caregiver, FGD, Akanran).*

*“People respect the VCHWs; this is more so because of the role they play relating to the health of children. That the VCHWs are lay people (non-formally trained health workers) like us does not make people look down on them in anyway anymore. Their low level of education does not affect the regard or respect we have for them. What makes them earn the respect from caregivers is that when a child is brought to them for treatment the child gets better” (Opinion leader, KII, Badeku, Male).*

The VCHWs themselves disclosed that the people well respected them due to the effective malaria treatment services they provide for under-five children in their communities. They disclosed that community members often refer to them as ‘nurses’ and ‘mothers of children’. Samples of their testimonies in this regard are contained in the following quotes:

*“Before we became VCHWs we were not different from other persons in the community. People in the community now have great respect for us after becoming VCHWs”* (VCHWs, FGD, Badeku).

*“We have been seen as special and people that should be respected. We are seen as Nurses in our communities, they give us great respect”* (VCHWs, FGD, Akanran).

*Community social support for the VCHWs*

A probe into the nature of social support provided by the people to the VCHWs was conducted. This did not yield any information relating to the provision of tangible or material support for the VCHWs. Rather support was reportedly provided in the form of “cooperation” with the VCHWs to ensure that the community-based childhood malaria treatment initiative was successful. Mention of “cooperation” as a form of social support cuts across the conducted FGDs and key informant interviews and this can be deduced from the following sample quotations:

*“People in our village (Jago) are cooperating with the VCHWs. This is being done so that the programme can continue”* (Male caregivers, FGD, Badeku).

*“People are cooperating with the VCHWs. We work hand-in-hand with them and caregivers take children to them for care” (TBA, KII, Badeku, Female).*

*Show of appreciation to the VCHWs*

The results revealed that community members were full of appreciation to the VCHWs for providing malaria treatment services. Elements of appreciation that cut across the VCHW themselves and opinion leaders in some of the communities are contained in the following verbatim expressions:

*“Community members celebrate the VCHWs. The fathers of most children brought to them for treatment go back to the VCHWs to show appreciation” (KII, Woman Leader, Badeku).*

*“After treating children, people come back to me the next day to thank me saying...we are grateful; our child is now well” (VCHW FGD Badeku).*

*“There was a day people took to the streets dancing and thanking VCHWs for what they are doing” (KII traditional leader, Jago, Male).*

## Discussion

The training, mobilisation, and supervision of VCHWs to be involved in recognising and managing childhood malaria within the context of PHC was an important intervention in the medically underserved rural agrarian communities. The training and involvement of laypersons as volunteer health workers in managing disease conditions within the context of PHC has a long history in Nigeria. For instance, community residents with little or no education have been trained and successfully used to control Onnchocerciasis^25^, provide medication counselling to patent medicine consumers^26^ and control malaria.^15^,^27^

However, there is a dearth of information on the use of volunteer non-health workers to provide evidence-based childhood malaria care involving the use of RDT in Nigeria. Before the childhood malaria treatment intervention in the study communities, only facility-based formal frontline health workers were involved in malaria management. Observations and informal discussions with the facility-based health workers revealed that treatment of malaria among various categories of people, including under-five children, was based on presumptive diagnosis; this was so because the scientific tools for the laboratory confirmation of malaria such as RDT and/or microscopes were not available.

To the residents in the study communities, using laypersons to diagnose and treat malaria using western medicine was a novelty; the initiative was, therefore, received with reservations or scepticisms at the initial stage. This response pattern to a new idea or practise is not uncommon; experiences dating to several decades ago have shown that innovations such as new practices are not usually adopted rapidly without reservations in all human populations. The factors which have potentials for influencing the adoption of innovations are related to the following: attitudes; degree of compatibility with cherished values; the relative advantage of what is being proposed for adoption over the existing practice or norm; simplicity or flexibility and the risks which may be associated with the innovation being introduced.^28^ These and other factors are useful for understanding why the use of VCHWs to treat malaria was characterised by elements of resistance, scepticism and a slow adoption process at the initial stage of the intervention. To address a phenomenon such as this, sustained public enlightenment, advocacy and social marketing of the under-five malaria treatment initiative is imperative. The use of testimonies relating to achievements being recorded by the VCHWs hold great potential for facilitating the reduction of barriers to the involvement of trained lay community-based health volunteers in the performance of health-related roles; this is more so when such roles are characterised by some elements of medical practice no matter how rudimentary.

Several factors account for the expression of respect for the VCHWs and satisfaction with their services. These factors include the following: the ready availability of anti-malaria medicines; easy access to and approachability of VCHWs; evidence-based diagnosis and provision of prompt and effective treatment free of charge. The qualities above also helped create a distinct niche for the VCHWs as dependable community-based volunteer health providers.

Studies have shown that these factors generally influence the utilisation of health services.^15^,^20^

The cooperation provided by the communities and the utilisation of the services provided by the VCHWs were perceived by community residents to be constituting a major form of support offered to ensure that the malaria treatment programme was successful. It is worthy of note that the “cooperation” alluded to in the results was in terms of acceptance of the VCHWs, respect for and willingness to work with them. The cooperation offered was also in the form of motivation and social incentives aimed at sustaining their services. Studies have earlier noted that cooperation expressed by community members in terms of trusting relationships and respect often have a positive influence on community health workers motivation and performance.^29^–^31^

It should be noted that the offered “cooperation” by the communities cannot, strictly speaking, be separated from the concept of community involvement which is always pivotal to the success of community-based health programme^32^. The communities were involved in nominating the laypersons for training as VCHWs guided by criteria that included honesty, integrity, permanent residents, and willingness to serve the community. In addition, the communities supported the VCHWs in kind by creating an enabling environment for them to work. On the other hand, the contributions of the team of investigators included the conduct of a training programme that focused on the treatment of childhood malaria; provision of health-related commodities; transport fare for VCHWs to participate in occasional refresher educational opportunities and supportive supervision.

## Conclusion

The community-based childhood malaria treatment programme facilitated by VCHWs was adjudged to be characterised by rewarding experiences by all the stakeholders. This unanimity of opinion is an indirect measure of the positive impact of the programme. The perceived success of the initiative indicates that laypersons can play key health-related roles in malaria management if they are well trained and guided.
